# Isolation of cholesterol-dependent, multidrug-resistant *Candida glabrata* strains from blood cultures of a candidemia patient in Kuwait

**DOI:** 10.1186/1471-2334-14-188

**Published:** 2014-04-08

**Authors:** Ziauddin Khan, Suhail Ahmad, Leena Joseph, Khaled Al-Obaid

**Affiliations:** 1 Department of Microbiology, Faculty of Medicine, Kuwait University, P. O. Box 24923, Safat 13110, Kuwait; 2 Microbiology Unit, Al-Amiri Hospital, Safat, Kuwait

**Keywords:** *Candida glabrata*, Drug resistance, Cholesterol auxotrophy, Candidemia

## Abstract

**Background:**

*Candida glabrata* has emerged as an important human pathogen associated with systemic and mucosal infections. Here, we describe isolation of two cholesterol-dependent *Candida glabrata* strains from a candidemia patient which failed to grow on the media devoid of a cholesterol source.

**Methods:**

Both the isolates were recovered from BACTEC Plus Aerobic/F blood culture bottles of a candidemic patient. Since these isolates failed to grow on Sabouraud dextrose agar, Mueller-Hinton agar and RPMI 1640 agar media, their definitive identification required PCR sequencing of the internally transcribed spacer (ITS)1 and ITS2 regions of rDNA and the D1/D2 region sequences within 26S rRNA gene. The cholesterol auxotrophy was determined by their ability to grow on media containing a cholesterol source. The minimum inhibitory concentrations (MICs) to antifungal agents were determined by Etest.

**Results:**

The identity of the isolates was confirmed by sequencing of the ITS1 and ITS2 regions of rDNA and the D1/D2 region sequences within 26S rRNA gene and also by matrix-assisted laser desorption and ionization–time-of-flight mass spectrometry with 99.9% confidence value. Both the isolates showed good growth only when media were supplemented with cholesterol, oxbile or blood. Additionally, these isolates were resistant to amphotericin B (MIC ≥32 μg/ml), fluconazole (MIC ≥256 μg/ml), voriconazole (MIC ≥32 μg/ml), itraconazole (MIC ≥32 μg/ml), and posaconazole (MIC ≥32 μg/ml), but susceptible to caspofungin (MIC range 0.064 to 0.19 μg/ml).

**Conclusion:**

This appears to be the first report on isolation of cholesterol-dependent strains of *C. glabrata* from a candidemia patient exhibiting resistance to azoles and amphotericin B. Further, the report demonstrates that induction of cholesterol/sterol auxotrophy is associated with resistance to antifungal drugs targeting ergosterol biosynthesis. These observations may have therapeutic implications for the treatment of infections caused by such *C. glabrata* strains.

## Background

*Candida glabrata* has emerged as the second most important yeast associated with mucosal and systemic infections in critically ill patients in some tertiary care hospitals in North America [1]. The species is intrinsically less susceptible to azoles and it is generally believed that extensive topical and systemic use of these drugs might have contributed to its rising incidence as a human pathogen. There are multiple mechanisms that impart resistance against azoles. These include alterations in *ERG11* gene that encodes for 14- α-methyl sterol demethylase in ergosterol biosynthesis and/or upregulation of efflux pumps encoded by *CgCDR1* and *CgCDR2* genes [2-4]. Additionally, alterations in *ERG3* gene encoding Δ^5,6^ sterol desaturase have been noted in strains carrying mutations in *ERG11* to allow survival under aerobic conditions [5]. Here, we describe the isolation of two *C. glabrata* strains from bloodstream of a candidemia patient which required exogenous cholesterol/sterol for growth in media that are routinely used in clinical mycology laboratories.

## Methods

### Isolation of *C. glabrata* strains

The isolates, Kw1018/12 and Kw1154/12, were obtained 9 days apart from two blood samples at Al-Amiri hospital, Kuwait. Initially, the isolates grew slowly in BACTEC Plus blood culture bottles with detection time of 54 and 75 hours, respectively and were tentatively identified by Vitek2 yeast identification system as *C. glabrata*. The isolates were received at Mycology Reference Laboratory, Faculty of Medicine, Kuwait University for further identification and antifungal susceptibility testing. When the isolates were subcultured on Sabouraud dextrose agar (SDA), they failed to grow. Similarly, no growth was obtained on RPMI medium with 2% glucose and Mueller-Hinton agar (MHA). Surprisingly, the isolates grew well on blood agar and chocolate agar. Addition of oxbile (2%), cholesterol (20 μg/ml) or sheep blood (5%) to SDA, MHA or RPMI supported good growth of the isolates.

### Molecular characterization of the isolates

Further species-specific identification of the cultured isolates was carried out by PCR sequencing of the internally transcribed spacer (ITS)1 and ITS2 regions of rDNA and the D1/D2 region sequences within 26S rRNA gene. Genomic DNA from the isolates was prepared and the ITS region of rDNA and D1/D2 regions of 26S rRNA gene were amplified and sequenced as described previously [6,7].

### PCR Sequencing of ERG11 and ERG3 genes

The complete *ERG11* gene (including 5′ and 3′ untranslated regions) was amplified by PCR using *rTth* DNA polymerase (Applied Biosystems, Foster City, CA, USA), ERG11F (5′-TCCACCTCGAACCCGTATA-3′) and ERG11R (5′-TCCATGTTGATATTCACGATGACT-3′) primers and by following the amplification and cycling conditions described previously [8]. The 1923 bp amplicons were sequenced by using Big-Dye terminator cycle sequencing kits (Applied Biosystems) and automated DNA sequencer 3130*xl* using ERG11FS1 (5′-GAACCCGTATACTCATCTCGTA-3′), ERG11FS2 (5′-GGTGATATCTTCTCTTTCATGCTA-3′), ERG11FS3 (5′-GACGTGAGAAGAACGATATCCA-3′), ERG11FS4 (5′-GTTACACTCACTTGCAAGAAGAA-3′), ERG11RS1 (5′-CACGATGACTTACTATTAGGCTAA-3′), ERG11RS2 (5′-CGAAACCGTAATCAACTTCGTCA-3′), ERG11RS3 (5′-ATCAAGACACCAATCAATAGGTT-3′), or ERG11RS4 (5′-AGTAAGCAGCTTCAGCGGAAACA-3′) as sequencing primer [8]. The complete *ERG3* gene (including 5′ and 3′ untranslated regions) was also amplified by PCR using *rTth* DNA polymerase, ERG3F (5′-AGAGATGAGGCCTGGAAGAAGA-3′) and ERG3R (5′-AAATATGAGAACCCAGGTCAGCA-3′) primers and by following the amplification and cycling conditions described previously [8]. The 1647 bp amplicons were sequenced by using ERG3FS1 (5′-GAAGAAGAGCTGATCTCTCTAGA-3′), ERG3FS2 (5′-TGTCTCTGAATAAGATCGTCTCT-3′), ERG3FS3 (5′-GTACGCCACTTTCATCTTCTTCA-3′), ERG3FS4 (5′-CCTGTTCGACCCTAAGCTAAAGA-3′), ERG3RS1 (5′-CAGGTCAGCACTTGAGTTTTCTCT-3′), ERG3RS2 (5′-GTCTTCTTCTTGTCGGTGTTGT-3′), ERG3RS3 (5′-CTTGTGCAAGGCCTTGTAGACA-3′), or ERG3RS4 (5′-CCTTGTTCACACGCTTCAAGGA-3′) as sequencing primer [8]. The complete ERG11 and ERG3 gene sequences were assembled and were compared with the corresponding sequences from reference *C. glabrata* strain 2001-L5 by using the program Clustal Omega (http://www.ebi.ac.uk/Tools/msa/clustalo/) [5].

### Antifungal susceptibility

Since both bloodstream *C. glabrata* strains failed to grow on RPMI medium, minimum inhibitory concentrations (MICs) were determined by Etest on RPMI medium containing 2% glucose and supplemented with cholesterol (20 μg/ml) and Mueller-Hinton agar (MHA) medium supplemented with cholesterol (20 μg/ml) or 5% sheep blood. For comparison, *C. glabrata* strain (ATCC 90030) and a recent blood culture isolate (Kw2273/13) were used as controls. The Etest method was performed using Etest strips (bioMérieux, Marcy-ĺEtoile, France) for fluconazole, itraconazole, voriconazole, caspofungin and amphotericin B. A standardized inoculum suspension of each isolate equivalent to a 0.5 McFarland standard was prepared. Plates were inoculated uniformly with cotton swab and allowed to dry before Etest strips were applied. MICs were determined after 48 h of incubation at 35°C. Azole MICs were read as the lowest concentrations producing an 80% reduction of growth. The isolates were considered resistant following the breakpoints described recently for *Candida* spp. [9].

Since these investigations were part of the routine diagnostic service offered by Mycology Reference Laboratory, no patient consent was required.

## Results

### Growth requirement

The isolates showed moderate to good growth on blood agar, chocolate agar and routine culture media supplemented with oxbile (2%), cholesterol (20 μg/ml) or sheep blood (5%).

### Molecular characterization

The complete ITS and D1/D2 region sequences of isolates Kw1018/12 and Kw1154/12 were assembled and the corresponding sequences from the two isolates were identical. In BLAST searches (http://www.ncbi.nlm.nih.gov/BLAST/Blast.cgi?), the ITS and D1/D2 region sequences of isolates Kw1018/12 and Kw1154/12 (EMBL accession nos. HE 993756/7 and HE 998775/6, respectively) showed only 7 and 1 nucleotide differences with the corresponding sequences from reference *C. glabrata* strain (CBS 138). The ITS region sequence is also available from another reference *C. glabrata* strain (ATCC 90030) and showed only 1 nucleotide difference with the corresponding sequences from isolates Kw1018/12 and Kw1154/12. Thus, the ITS and/or D1/D2 region sequences of both the isolates exhibited >99% sequence identity with the corresponding sequences from the reference *C. glabrata* strains confirming their identity as *C. glabrata.* The species-specific identity of the isolates was also confirmed by matrix-assisted laser desorption and ionization–time-of-flight mass spectrometry (MALDI–TOF MS; bioMeriuex) with 99.9% confidence value.

The *ERG11* gene sequences of our isolates (EMBL accession number HF952117) showed four synonymous mutations within the coding region compared to the sequence from reference *C. glabrata* strain 2001-L5 [5]. Similarly, the *ERG3* gene sequences of our isolates (EMBL accession number HF952118) showed three synonymous mutations within the coding region compared to the sequence from reference *C. glabrata* strain 2001-L5. No non-synonymous mutations were detected and no promoter mutations were apparent in both *ERG11* and *ERG3* genes.

### Antifungal susceptibility

**Figure 1:**
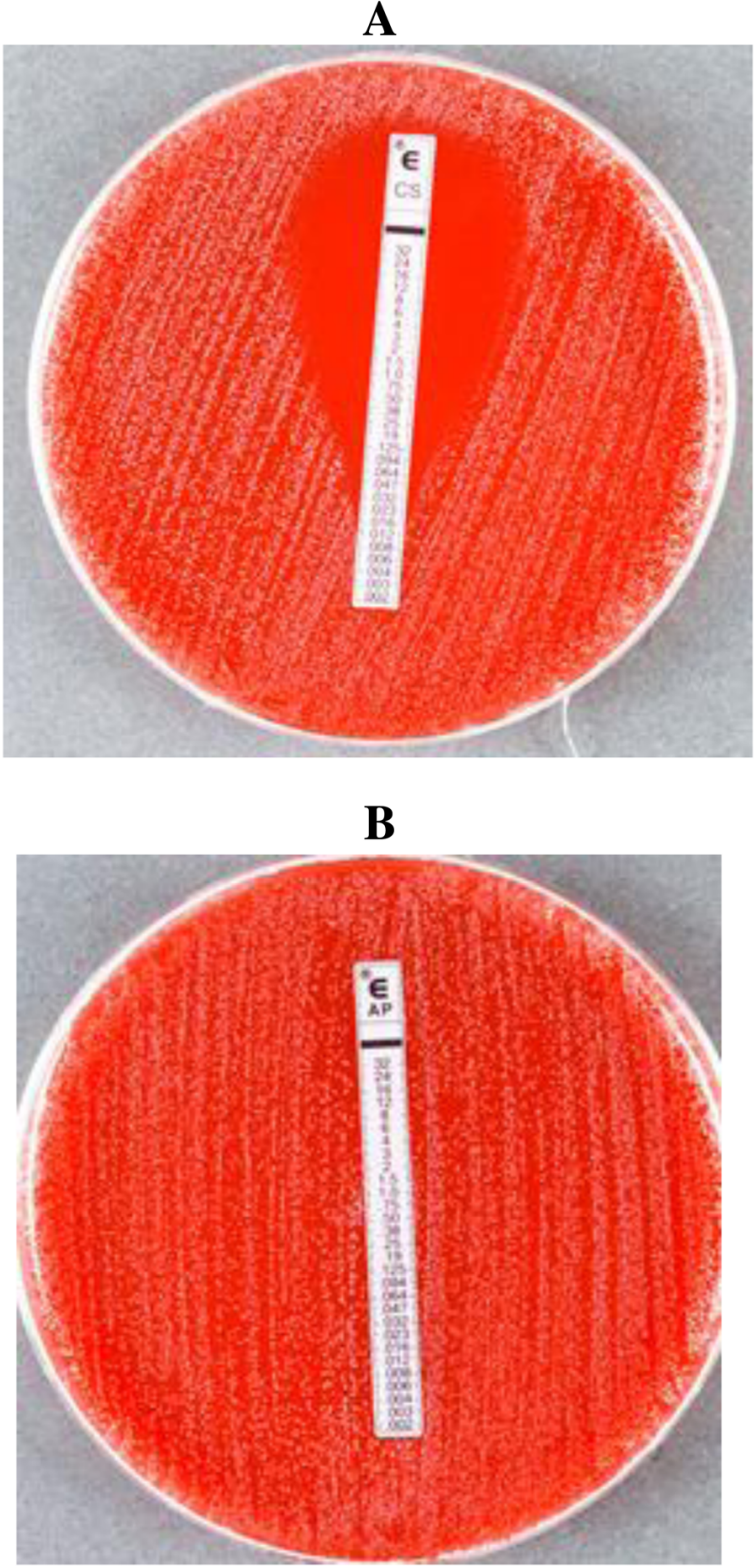
***Candida glabrata *** **strain (Kw1018/12) showing Etest MICs for caspofungin (CS, 0.094 μg/ml) (A) and amphotericin B (AP, >32 μg/ml) (B) on Mueller-Hinton agar supplemented with 5% sheep blood and read after 48 h of incubation at 35°C.**

Both our auxotrophic *C. glabrata* strains were resistant to amphotericin B (MIC ≥32 μg/ml), fluconazole (MIC ≥256 μg/ml), voriconazole (MIC ≥32 μg/ml), itraconazole (MIC ≥32 μg/ml), and posaconazole (MIC ≥32 μg/ml), but appeared susceptible to caspofungin (MIC range 0.064 to 0.19 μg/ml) on all the media used (Figure 1, Table 1) [9]. In contrast, the MICs of control strain of *C. glabrata* (ATCC 90030) as well as of a recent blood culture isolate (Kw2273/13) were within susceptible range (Table 1).

**Table 1:** **Comparative antifungal susceptibility of *C. glabrata *** **strains on different media with and without cholesterol auxotrophy**

**Strain No**	**Minimum inhibitory concentration (μg/ml) on**																							
	**RPMI with 2% glucose**						**RPMI with cholesterol (20 μg/ml)**						**MHA with cholesterol (20 μg/ml)**						**MHA with 5% sheep blood**					
	**AP**	**FL**	**VO**	**IT**	**PO**	**CS**	**AP**	**FL**	**VO**	**IT**	**POS**	**CS**	**AP**	**FL**	**VO**	**IT**	**POS**	**CS**	**AP**	**FL**	**VO**	**IT**	**POS**	**CS**
Kw1018/12	-	-	-	-	-	-	≥32	≥256	≥32	≥32	≥32	0.125	≥32	≥256	≥32	≥32	≥32	0.19	≥32	≥256	≥32	≥32	≥32	0.094
Kw1154/12	-	-	-	-	-	-	≥32	≥256	≥32	≥32	≥32	0.125	≥32	≥256	≥32	≥32	≥32	0.19	≥32	≥256	≥32	≥32	≥32	0.064
Kw2273/13	0.38	8	0.25	6	2	0.094	1	4	0.125	4	2	0.125	1	3	0.19	6	0.75	0.25	0.38	3	0.19	1	0.5	0.094
ATCC 90030	0.19	6	0.5	8	4	0.094	0.5	3	0.064	6	4	0.125	0.38	6	0.25	3	6	0.19	0.38	3	0.094	0.5	0.25	0.094

## Discussion

A literature review revealed that some strains of *C. glabrata* require exogenous supply of cholesterol for their growth [10-12]. Both our blood culture isolates grew on blood agar and chocolate agar, but exhibited growth on SDA, RPMI agar or MHA only when supplemented with oxbile (2%), cholesterol (20 μg/ml) or sheep blood (5%). Isolation of cholesterol-requiring *C. glabrata* strains from candidemia patients may not be difficult since patient’s blood can serve as a source of cholesterol. However, *C. glabrata* strains requiring exogenous cholesterol for growth have also been recovered from non-blood specimens and were found to be resistant to antifungal drugs that act on ergosterol biosynthesis [11,12]. Thus, patients infected with such strains may pose significant diagnostic and therapeutic challenges because of lack of growth on routine culture media, but will exhibit *in vivo* growth due to their ability to maintain integrity of cell membrane by utilizing exogenous cholesterol available in blood/tissue milieu. Consequently, such strains can persist at the site of infection in spite of adequate therapy with azoles or amphotericin B. Although cholesterol-dependent strains of *C. glabrata* are rarely encountered, it is possible that isolation of some such strains might be missed if specimens are cultured only on routine media (devoid of a cholesterol source). Here, it is pertinent to mention that *C. glabrata* is prone to developing reduced susceptibility/cross-resistance to multiple antifungal agents without cholesterol/sterol auxotrophy [13-15]. The development of cholesterol auxotrophy seemingly does not affect susceptibility to caspofungin or other echinocandins, which is the treatment of choice for *C. glabrata* infections [16].

The patient from whom the two isolates (Kw1018/12 and Kw1154/12) were recovered was initially treated with liposomal amphotericin B (AmBisome, 5 mg/kg) for three weeks and later with caspofungin (70 mg loading dose, followed with 50 mg daily dose) for 7 days. However, the patient died of multi-organ failure and septic shock. It is probable that the appropriate therapy with caspofungin was delayed in our patient until *C. glabrata* was isolated and its resistance against amphotericin B and azoles was determined.

The major mechanisms known to mediate resistance to azoles in *C. glabrata* involve upregulation and/or other mutations in *ERG11* and efflux pumps. However, resistance to azoles in our isolates was not due to mutations in *ERG11* gene and non-synonymous mutations were also not detected in the *ERG3* gene. Although the nucleotide sequences of both genes varied slightly, no promoter mutations were detected and the encoded protein sequences (EMBL accession numbers HF952117 and HF952118) were also identical to reference *C. glabrata* strain 2001-L5 [5]. Upregulation of efflux pumps was not studied in the present investigation. Thus, the molecular basis of resistance to azoles in our isolates remained unidentified. It is probable that upregulation of efflux pumps or mutation(s) in some other genes that have been recently shown to confer resistance to antifungal drugs in *C. glabrata* are involved in our isolates [3,17-19]. Occurrence of such strains poses a major threat for proper management of such patients, particularly when resistance to echinocandins among *C. glabrata* strains is also emerging rapidly [20].

## Conclusion

Two cholesterol-dependent *C. glabrata* strains isolated from the blood of a candidemic patient are described. The report demonstrates that induction of cholesterol/sterol auxotrophy in *C. glabrata* may impart resistance to antifungal drugs targeting ergosterol biosynthesis. The observations may have therapeutic implications. Early therapy with echinocandins may be optimal to overcome the problem of triazole/amphotericin B resistance in such isolates. A limitation of our study is that the molecular basis of resistance to azoles in our isolates remained unidentified as only two genes involved in ergosterol biosynthesis were studied while efflux pumps that can also contribute towards resistance to azoles were not investigated. To our knowledge, this is the first report on the isolation of cholesterol-dependent strains of *C. glabrata* from a candidemia patient exhibiting resistance to azoles and amphotericin B.

## Abbreviations

AP: Amphotericin B; FL: Fluconazole; VO: Voriconazole; IT: Itraconazole; POS: Posaconazole; CS: Caspofungin.

## Competing interests

The authors declare that they have no competing interests.

## Authors’ contributions

ZUK and SA drafted the manuscript. LJ performed antifungal susceptibility and molecular identification studies. KA collected the clinical data. All authors read and approved the final version of the manuscript.

## Pre-publication history

The pre-publication history for this paper can be accessed here:

http://www.biomedcentral.com/1471-2334/14/188/prepub
